# Cholera outbreak: antibiofilm activity, profiling of antibiotic-resistant genes and virulence factors of toxigenic *Vibrio cholerae* isolates reveals concerning traits

**DOI:** 10.1099/acmi.0.000324

**Published:** 2022-03-23

**Authors:** Silas O. Awuor, Eric O. Omwenga, Richard M. Mariita, Ibrahim I. Daud

**Affiliations:** ^1^​ School of Health Sciences, Kisii University, P.O BOX 408-40200 Kisii, Kenya; ^2^​ Microbial BioSolutions, Troy, New York, 12180, USA; ^3^​ Kenya Medical Research Institute, United States Army Medical Research Directorate-Africa, HJF Medical Research International, Kericho, Kenya

**Keywords:** antibiofilm activity, antibiotics, resistant genes profiling, *Vibrio cholerae*, virulence factors

## Abstract

*
Vibrio cholerae* is a biofilm-forming pathogen with various virulence phenotypes and antimicrobial resistance traits. Phenotypic characteristics play a critical role in disease transmission and pathogenesis. The current study elucidated antibiofilm formation activity, profiled antibiotic-resistant genes and virulence factors of toxigenic *Vibrio cholerae* isolates from the cholera outbreak in Kisumu County, Kenya. *Vibrio cholerae* O1 isolates collected during the 2017 cholera outbreak in Kisumu County, Kenya, were utilized. Biofilm and virulence factors were profiled using standard procedures. The study confirmed 100 isolates as *Vibrio cholerae *, with 81 of them possessing cholera toxin gene (*ctxA*). Additionally, 99 of the isolates harboured the *toxR* gene. The study further revealed that 81 and 94 of the isolates harboured the class I integron (encoded by *inDS* gene) and integrating conjugative element (ICE), respectively. Antibiotic resistance assays confirmed tetracycline resistance genes as the most abundant (97 isolates). Among them were seven isolates resistant to commonly used antibiotics. The study further screened the isolates for antibiofilm formation using various antibiotics. Unlike the four strains (*03/17–16, 02/17–09, 04/17–13*), three of the strains (*04/17–07, 06/17–14* and *05/17–03*) did not form biofilms. Further, all the seven isolates that exhibited extensive antibiotic resistance produced haemolysin while 71.42%, 85.71 and 71.42 % of them produced protease, phospholipases and lipase, respectively. This study provides and in-depth understanding of essential features that were possibly responsible for *V. cholerae* outbreak. Understanding of these features is critical in the development of strategies to combat future outbreaks.

## Introduction

*Vibrio cholerae* is a causative agent of cholera, an acute diarrheal infection caused by ingestion of contaminated food or water. *Vibrio cholerae* belongs to genus *vibrio*, family *Vibrionaceae* [1]. There are two biotypes of *Vibrio cholerae,* classical and El Tor. The two biotypes differ in severity of clinical symptoms, as well as expression and regulation of major virulence factors [2]. *Vibrio cholerae* has several factors, which help reach and colonize the epithelium of the small intestine, leading to the production of a variety of extracellular products that have deleterious effects to eukaryotic cells [3].

Cholera has unique epidemiology features, the intriguing ones being the predisposition to cause epidemics with pandemic potential and the ability to remain endemic in all affected areas [4]. The bacteria are transmitted between humans through the faecal–oral route; a bite of contaminated food or a sip of contaminated water can cause infection [5]. Globally, the burden of cholera is highest in sub-Saharan Africa where between 2012 and 2015, six pandemics of cholera have been recorded [6]. In 2016 alone, 132 121 cholera cases with 2420 deaths were reported to the World Health Organization (WHO) worldwide. Overall, 13 % of cases were reported from the USA, 17 % from Asia, 45 % from Africa and 25 % from Kenya [7].

The two major virulence factors expressed by *V. cholerae* O1 and O139 are cholera toxin (CT), an AB5 family ADP-ribosyltransferase that is responsible for the profuse rice-watery diarrhoea typical of this disease [2], and the toxin-coregulated pilus (TCP), a type IV pilus that mediates adherence and microcolony formation and is required for intestinal colonization in neonate mice and humans [8, 9]. The genes encoding the CT subunits ctxA and ctxB constitute an operon within the prophage form of the filamentous phage CTXF [10]. The genes required for TCP biogenesis form a large cluster known as the *V. cholerae* pathogenicity island (VPI) or TCP island [11]. Within this cluster, tcpA encodes the major pilus subunit [12].

Other *V. cholerae* genes are corregulated in the same manner including the tcp operon, which is responsible for fimbrial synthesis and assembly. The ctx operon and the tcp are part of regulon, whose expression is controlled by the same environmental signals [13]. The proteins involved in the control of this regulon expression have been identified as ToxR, ToxS and ToxT. ToxR is a trans membranous protein with about two thirds of its amino terminal part exposed to the cytoplasm. ToxS is a periplasmic protein. It is thought that ToxS can respond to environmental signals, change conformation and can influence dimerization of ToxR, which activates transcription of the operon. Expression of ToxT is activated by ToxR, while ToxT in turn activates transcription of tcp genes for synthesis of tcp pili [14].

These factors included other potential toxins, accessory colonization factors, outer membrane proteins, proteases, haemolysins, haemagglutinins (HAs), and in some strains, a capsular polysaccharide, all of which may contribute to survival and multiplication within the host [10, 15]. The genes that encode the cholera toxin subunits ctxA and ctxB are localized at a CTX genetic element, which is made up of a 4.6 kbp central core region 2.4 kbp repititive sequence termed RS2. Similar RS sequences called RS1 may flank the CTX element [16]. Zonula occludens toxin increases the permeability of enterocytes and is encoded by *zot* gene, which is within the core region of CTXф [17]. A third toxin encoded by *ace* affects intestinal secretion [16]. HylA for haemolysin damages cells by acting as pore-forming toxin and studies have shown purified haemolysin is enterotoxic [10]. Outer membrane proteins, which include *OmpU*, *OmpT*, *OmpS*, *OmpV* and others, are major cell-envelope proteins. *OmpU* is thought to contribute to bile resistance and functions as a colonizing factor [18]. Bile salts facilitate survival of *V. cholerae* in the intestine [19]. Toxin co-regulated pilus is clearly required for intestinal colonization [20].

**Fig. 1.**
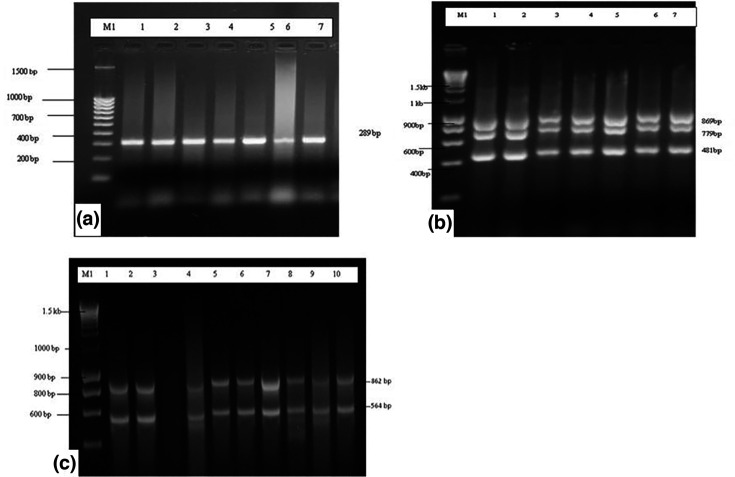
Gel electrophoresis for confirmation of various genes extracted from the seven isolates of *V. cholerae* that were resistant to various commonly used antibiotics with plate A representing the *ctxA* gene in clinical *V. cholerae *, plate B representing *inDS, toxR* and *int* genes in clinical *V. cholerae* isolate and plate C representing *tetA* and *Ery* genes in clinical *V. cholerae* isolates. The legends: lane 1 (05/17–03 isolate^*^); lane 2 (06/17–14 isolate^*^); lane 3 (03/17–16 isolate^*^); lane 4 (04/17–07 isolate^*^); lane 5 (02/17–09 isolate^*^); lane 6 (04/17-13Isolate^*^) and lane 7 (06/17–07 isolate^*^). Lane M1 for plate A represents 2 kb DNA Size Marker- Hyper ladder I *ctxA* band 289 bp; lane M1 for plate B represents 2 kb DNA Size Marker- Hyper ladder I *inDS* 869 bp, *toxR* 779 bp and *int* 481 bp and lane M1 for plate C represents 2 kb DNA Size Marker- Hyper ladder I, *tetA* 862 bp and *Ery* 564 bp. * The figures in brackets represent strain numbers.

The *V. cholerae* biofilm life cycle occurs in four stages (graphical Fig. 1), starting with the initial attachment of cells to a surface, formation of microcolonies, maturation of the microcolonies into an established biofilm, and dispersal of planktonic bacteria from the biofilm [19].

In all these phases of biofilm formation, quorum-sensing (QS) system is involved in the regulation of population density and metabolic activities. QS system is a central component of bacterial cell-to-cell communication [21], acting as a language for the interaction among the neighbouring bacteria that collectively and genetically respond to the extracellular, diffusible small molecule signals released in a cell-density-dependent manner [22]. As such, the production of molecule signals – autoinducing peptides (AIP) such as AgrD peptide – can be controlled and helps the bacteria in overwhelming the host defences by secreting exotoxins after sufficient colonization in the host has taken place [23].

Microbial cells embedded in a matrix containing polysaccharides, proteins and extracellular microbial DNA are known as biofilm [24–26]. The biofilm-forming pathogens like *Vibrio cholerae* and drug-resistant pathogens like MRSA can cause fatal diseases to human beings and become resistant to most of the antimicrobial drugs [27]. This is because biofilms are less susceptible to antibiotics, and provide a reservoir for microbial cells, which when dispersed enhances the risk of chronic and persistent infections. It may also promote the reinfection of colonized sites hence increasing the management cost and hospital stay [28, 29]. Likewise, the matrix confers a protection against biocides, immune system activity and drugs and has environmental promoters that induce biofilm formation that contributes to drug resistance development [30, 31]. All these factors contribute to biofilm cells being 1000-fold more resistant to antimicrobial agents than planktonic cells [24]. Moreover, treatment and control of biofilm is complicated because the current antimicrobials have been developed for planktonic cells that are metabolically active [32].

Other virulence factors are also produced by different strains of *V. cholerae* and may be related to the hydrolysis of lipid barrier in intestinal epithelial cells [33]. While Neuraminidase, is secreted to cause an increase in the number of receptors in the gut [34]. Therefore, it is against this background that this study focused on deducing biofilm formation and inhibition ability, virulence and resistant gene profiling of the *V. cholerae* isolates obtained from the 2017 cholera outbreak at Kisumu County, Kenya. An in-depth understanding of essential features of *V. cholerae* strains involved in cholera outbreaks will help in the development strategies to combat future outbreaks.

## Methods

### (i) The *V. cholerae* isolates' source

A total of 119 isolates were obtained from the Kenya medical research Institute (KEMRI – Kisumu) where cultures stocks have been kept following the 2017 cholera outbreak at −80 °C. Long-term storage of specimens at −80 °C was done in 10 % glycerol. These cultures were obtained from stool samples randomly selected from patients with severe diarrhoea suspected of having cholera during the outbreak. Consent was obtained from the patients prior to collection of stool samples.

### (ii) Culturing of the *V. cholerae* isolates

Diarrhoeal stool samples were thawed and cultured on alkaline peptone water followed by 6 h incubation at 37 °C using established procedures as used before [35]. The cultures were then plated on thiosulfate citrate bile salts sucrose (TCBS) agar following the manufacturer’s instructions. Agar plates were incubated at 37 °C for 18–24 h. Typical yellow colonies, which were presumed to be *V. cholerae* isolates [36], were selected and subjected to biochemical, serological and genotypic analyses as previously outlined [35].

### (iii) Biofilm formation inhibition assay

As described by Omwenga *et al*. [37], microtitre plate assay was performed to quantify the effect of commonly used antibiotics on the biofilm formation of *V. cholerae* strains. The test bacteria were first inoculated on Luria-Bertani medium (LB) agar and incubated at 37 °C overnight. Then a colony was identified, picked and inoculated in 10 ml of LB broth and incubated at 37 ˚C overnight while shaking at 100 r.p.m. for 18 h. By use of a parafilm the flat-bottomed polystyrene tissue culture microplate was sealed for purposes of preventing medium evaporation. After 48 h incubation, the wells were carefully rinsed with double-distilled water to remove loosely attached cells. The microplate was air-dried for 1 h before adding 200 µl per well of 0.4 % crystal violet (CV) solution to the adhered cells in the wells and then stand at room temperature for 15 min. Excess stain was removed by rinsing the wells gently with 200 µl per using distilled water. This was repeated thrice. The microtitre plate was then air-dried for 1 h after, followed by addition of 200 µl of absolute ethanol to each well to solubilize the dye (Data S1, available in the online version of this article). The OD was measured at OD_590_nm using a Safire Tecan-F129013 Microplate Reader (Tecan, Crailsheim, Germany). For each experiment, background staining was corrected by subtracting the crystal violet bound to un-treated controls (Blank) from those of the tested sample. The experiments were done in triplicate and average OD_590_nm values were calculated. To estimate the antibiofilm activity (Abf A) of a given antibiotic the following equation was used

Abf A (%) = (1-(OD_Test sample_ - OD_Blank_)/ (OD _Untreated sample_ – OD _Blank_)×100.

### (iv) Molecular biology techniques/assays – PCR and gel electrophoresis

Confirmation of identification of *V. cholerae* was performed using a conventional PCR assay targeting the ctxA gene. Additionally, *Vibrio* spp. were screened for the presence of *CtxA, toxR, inDS, int, tetA* and *Ery* gene to determine the specificity of the assay. The specific primers (Data S2) selected for PCR analysis of the *ompW* gene, according to [3]. These primers are synthesized by the Alpha DNA Company, Canada. PCR analysis was conducted in 50 µl of a reaction mixture containing 24 µl of GoTaq Green Master, 2 µl of 25 mmol l^−1^ MgCl_2_, 2 µl of (100 pmol) primer, and 10 µl of distilled water. Amplification was conducted using a master cycler (Eppendorf) programmed with 1 cycle at 95 °C for 1 min, 40 cycles of 95 °C for 1 min, 64 °C for 1 min, 72 °C for 1 min, 72 °C for 10 min. The amplified product was subjected to 1.8 % agarose gel electrophoresis and visualized under UV after ethidium bromide staining [8].

### (v) Detection of other virulence factors of *V. cholerae*

### Detection of haemolysin

Haemolysin production by the *V. cholerae* isolates was detected following protocols by Benson *et al*. [38]. The β-haemolytic activity was tested for on base agar (Himedia, India) supplemented with 7 % sheep erythrocytes for 18–24 h. Pure isolates were cultured on TSA, before streaking on blood agar and further incubated for 24 h at 37 °C. Zones of haemolysis around the colonies indicated the ability of these bacteria to haemolyse RBCs [39].

### Detection of protease

To detect protease production by the *V. cholerae* isolates skim milk agar was used and the protocol that was described in [38]. Briefly, two solutions (A and B) were made and used in this study. Solution A was prepared by adding 10 g skim milk to 90 ml of distilled water then volume was completed to 100 ml gently heated at 50 °C, then autoclaved and cooled to 50–55 °C. And solution B was also prepared by adding 2 g of agar powder to 100 ml of distilled water, mixed thoroughly, then autoclaved and cooled to 50–55 °C. Aseptically, 100 ml of solution A was mixed with 100 ml of solution B. Then the mixture was poured into sterile petri dishes, and then stored at 4 °C until use. This media used to detect the ability of the bacteria to produce protease [30]. The appearance of a cleared hydrolysis zone indicates a positive test [39].

### Detection of lipase

Lipase production ability by *V. cholerae* isolates was determined by methods outlined by Elliot *et al*. [39]. Briefly, a single colony of an overnight growth was cultured on Rhan medium (Data S3), and then incubated for 1–5 days at 37 °C. The appearance of a turbid zone around colonies indicates a positive result [38].

### Detection of lecithinase (phospholipase)

To detect lecithinase, we followed a standard procedure [40]. One pure colony was cultured on medium of phospholipase activity assay (Data S4) followed by incubation for 1–3 days at 37 °C using established procedures [35]. The appearance of a white to brown colour elongated precipitated zone around colonies is considered a positive result [1].

### Statistical analysis

Statistical analyses were carried out using Graph pad prism (version 8; La Jolla, CA, USA). Means were presented as mean±sem as all tests were carried out in triplicates.

## Results

### *V. cholerae* O1 virulence genes

Eighty-one (80.2 %) isolates possessed the cholera toxin gene (*ctxA*). Analysis of the *toxR* gene revealed that 99 (98.0 %) harboured the *toxR* gene. Using PCR, a variety of pathogenic and antimicrobial resistance genes were detected (Table 1). It was also revealed that 81 (80.2 %) of the isolates harboured the class I integron (encoded by *inDS* gene). The majority, 94 (93.1 %) were confirmed to possess the SXT integrating conjugative element (ICE). The tetracycline resistance gene was present in 97 (96.0 %) of the isolates. Seven isolates were confirmed to be resistant against commonly used antibiotics.

**Table 1.** Analysis of pathogenic and antimicrobial resistance genes (Data S3) by PCR in *V. cholerae* isolates from cholera outbreaks in Kisumu County, 2017 (*n*=101)

Primer	Target gene	Positive *N* (%)	Negative *N* (%)
ctxA	Cholera toxin	81 (80.2)	20 (19.8)
toxR	Regulatory gene	99 (98.0)	2 (2.0)
inDS	Class one intergron	81 (80.2)	20 (19.8)
int	SXT intergrase	94 (93.0)	7 (7.0)
tetA	Tetracycline resistance	4 (4.0)	97 (96.0)
Ery	Erythromycin resistance	90 (90.0)	11 (10.0)

Based on PCR analysis, the *ace* gene was revealed in all seven isolates as shown in plate A (Fig. 1). Also, *inDS, toxR* and *int* genes were revealed to be present as presented in plate B (Fig. 1). Additionally, PCR genotyping also did show the presence of *ctxA* and *tcpI* genes from clinical *V. cholerae* isolates as represented in plate C in Fig. 1 above.

### Biofilm formation inhibitory effects of selected antibiotics against the *V. cholerae* strains

**Fig. 2.**
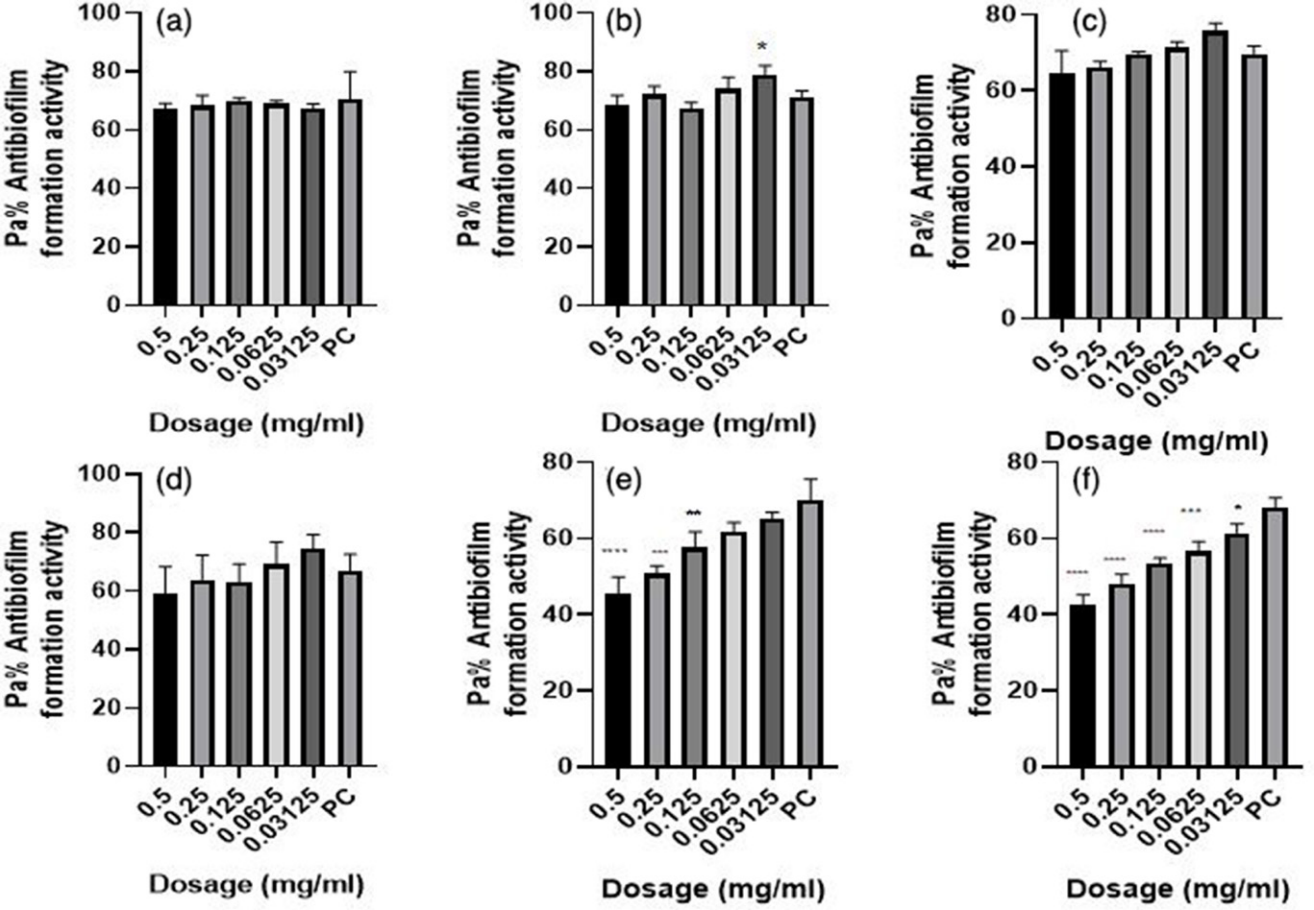
Antibiofilm formation activity against isolate 03/17–16 of *V. cholerae* against various antibiotics: (a) tetracycline (b) ampicillin, (c) amoxicillin, (d) cotrimoxazole, (e) erythromycin and (f) nalidixic acid; PC=*P. aeruginosa* – Positive control (*n*=3, ANOVA Dunnett’s multiple comparisons test; *P=0.05; **P=0.01; ***P=0.001; ****P=0.0001).

**Fig. 3.**
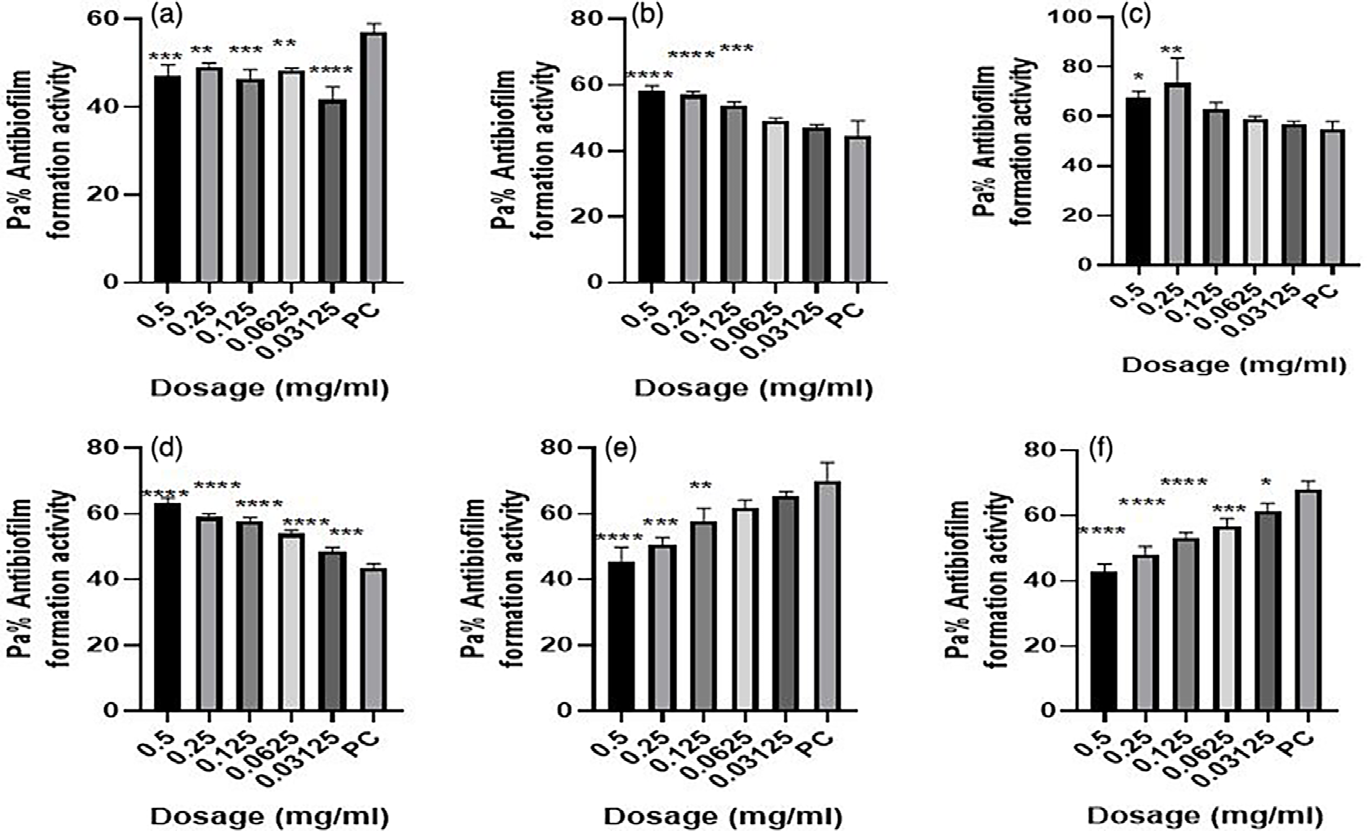
Antibiofilm formation activity against isolate 02/17–09 of *V. cholerae* against various antibiotics: (a) tetracycline, (b) ampicillin, (c) amoxicillin, (d) cotrimoxazole, (e) erythromycin and (f) nalidixic acid; PC=*P. aeruginosa* – positive control (*n*=3, Anova Dunnett’s multiple comparisons test; *P=0.05; **P=0.01; ***P=0.001; ****P=0.0001).

**Fig. 4.**
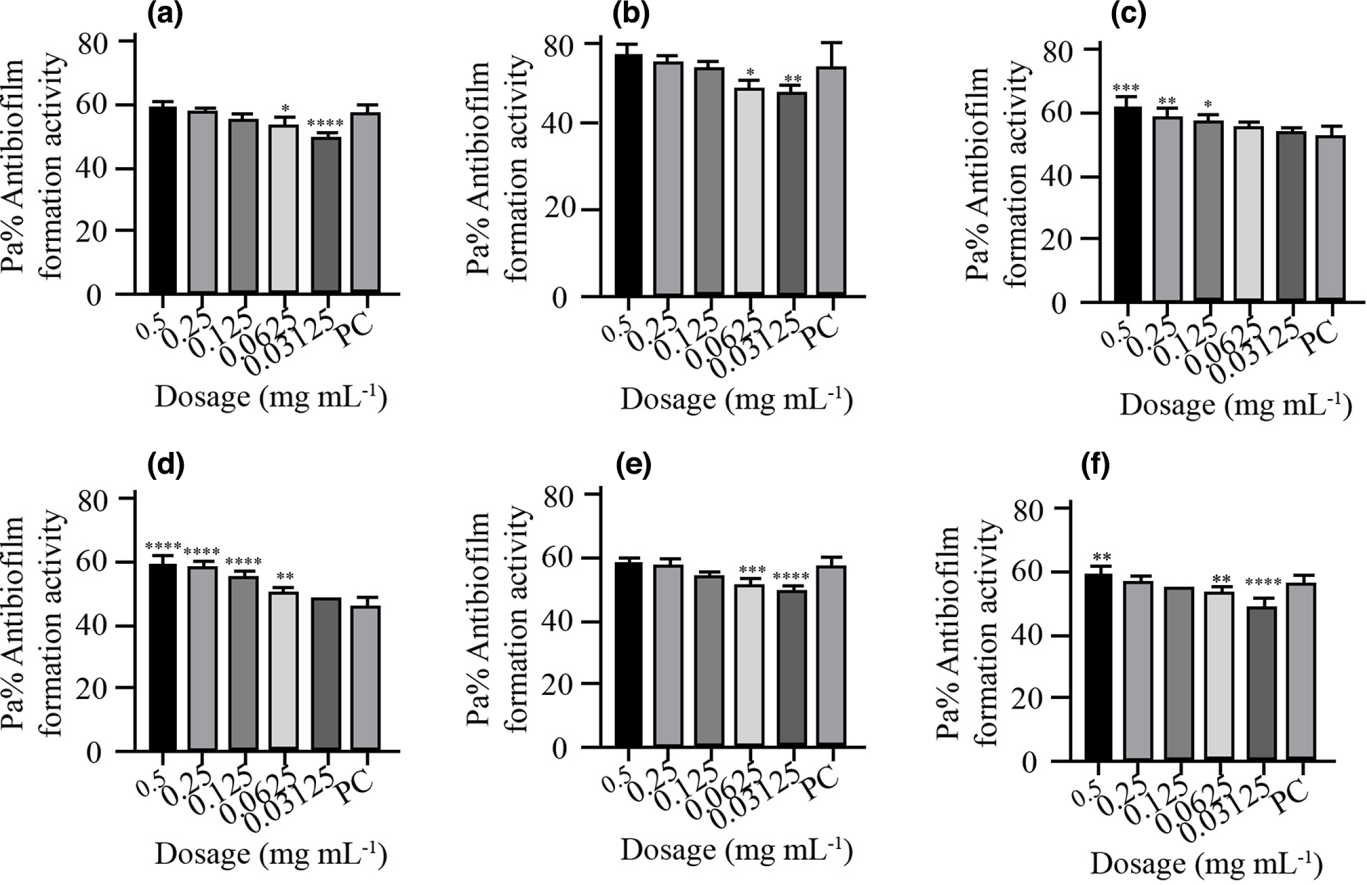
Antibiofilm formation activity against isolate 04/17–13 of *V. cholerae* against various antibiotics: (a) tetracycline (b) ampicillin, (c) amoxicillin, (d) cotrimoxazole, (e) erythromycin and (f) nalidixic acid; PC=*P. aeruginosa –* positive control (*n*=3, Anova Dunnett’s multiple comparisons test; *P=0.05; **P=0.01; ***P=0.001; ****P=0.0001). Against this test isolate 04/17–13, inhibitory activities were observed to be more at lower dosages than at high dosages.

**Fig. 5.**
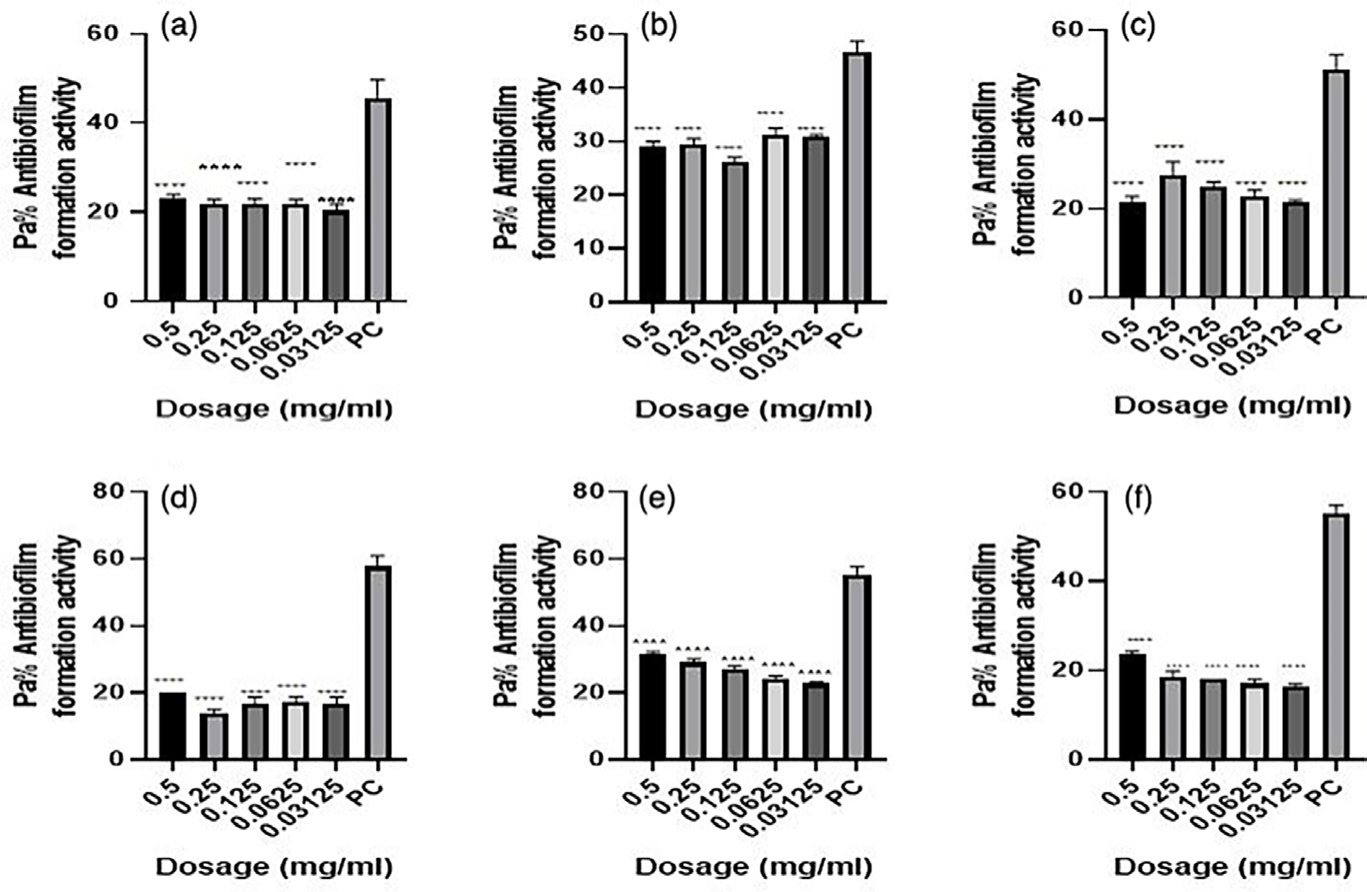
Antibiofilm formation activity against isolate 05/17–07 of *V. cholerae* against various antibiotics: (a) tetracycline (b) ampicillin, (c) amoxicillin, (d) cotrimoxazole, (e) erythromycin and (f) nalidixic Acid; PC=*P. aeruginosa* – positive control (*n*=3, Anova Dunnett’s multiple comparisons test; *P=0.05; **P=0.01; ***P=0.001; ****P=0.0001).

**Fig. 6.**
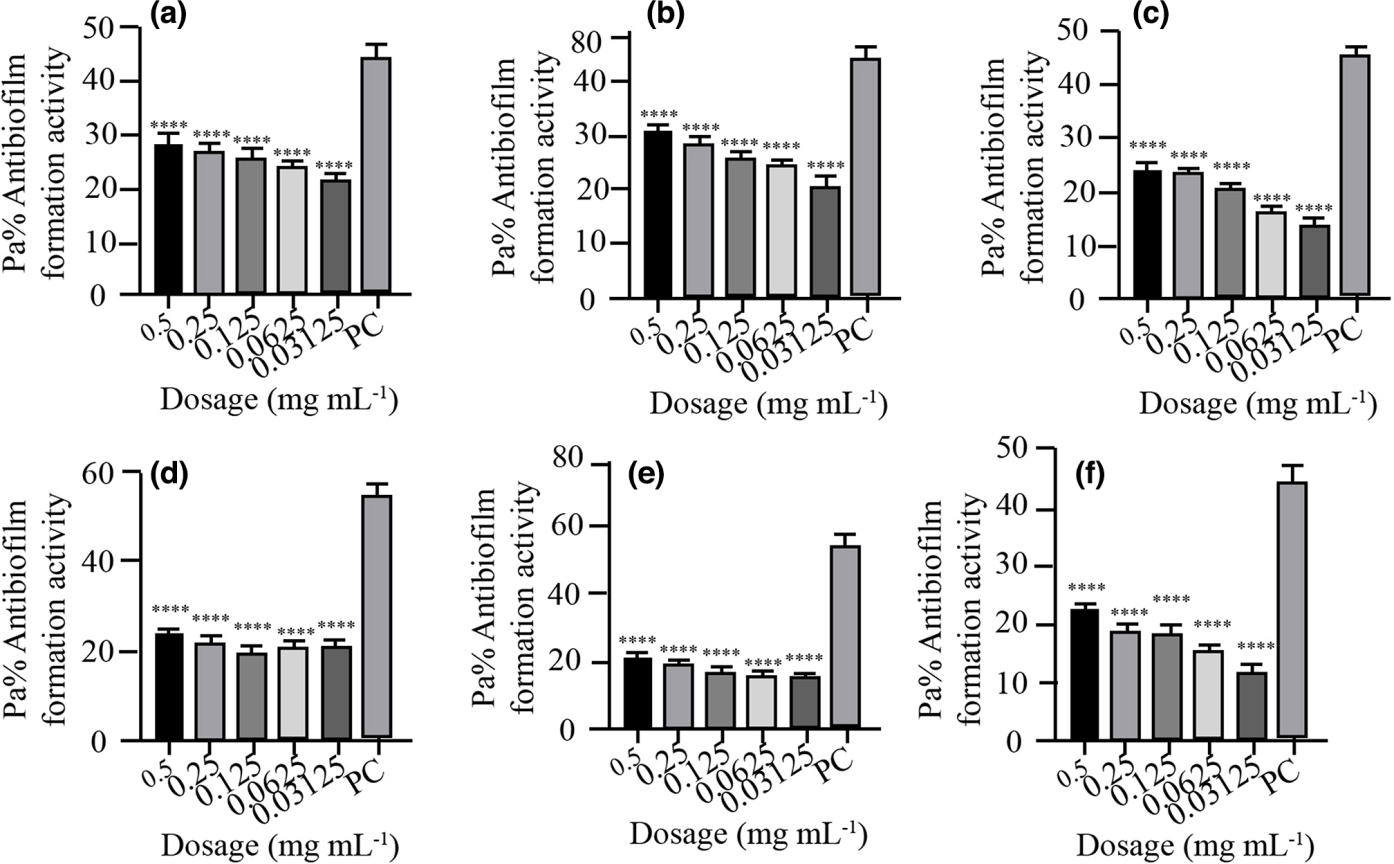
Antibiofilm formation activity against isolate 05/17–03 of *V. cholerae* against various antibiotics: (a) tetracycline, (b) ampicillin, (c) amoxicillin, (d) cotrimoxazole, (e) erythromycin and (f) nalidixic acid; PC=*P. aeruginosa* – positive control (*n*=3, Anova Dunnett’s multiple comparisons test; *P=0.05; **P=0.01; ***P=0.001; ****P=0.0001).

**Fig. 7.**
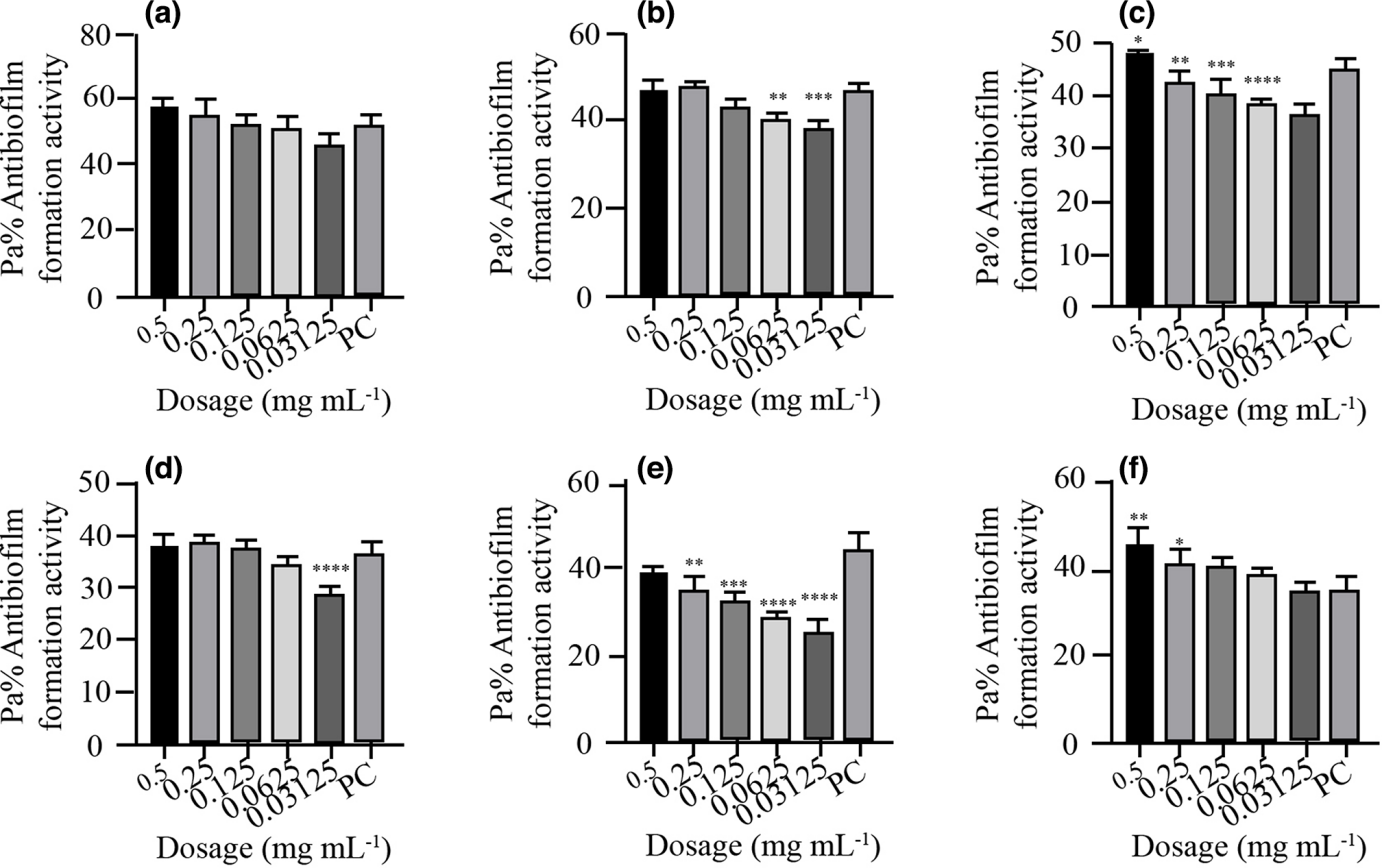
Antibiofilm formation activity against isolate 06/17–14 of *V. cholerae* against various antibiotics: (a) tetracycline, (b) ampicillin, (c) amoxicillin, (d) cotrimoxazole, (e) erythromycin and (f) nalidixic acid; PC=*P. aeruginosa –* positive control (*n*=3, Anova Dunnett’s multiple comparisons test; *P=0.05; **P=0.01; ***P=0.001; ****P=0.0001).

The most resistant drugs towards the seven isolates (tetracycline, ampicillin, amoxicillin, cotrimoxazole, erythromycin and nalidixic acid) [36] were used as treatments for the antibiofilm formation assay in a 96-well microtitre plate. Different concentrations of the drugs were prepared using twofold serial dilution (with dosage ranging from 0.5 to 0.03125 mg ml^−1^) and the wells were inoculated with 10 µl of *V. cholerae* isolates. *P. aeruginosa* ATCC10145 was used as positive control. The results showed that the biofilm formation inhibitory effects of the various concentrations (0.5, 0.25, 0.125, 0.0625 and 0.03125 mg ml^−1^) were significantly lower than that of the positive control, an indication that biofilm formation was inhibited at these concentrations (Figs. 2–7). As much as such inhibitory effects were recorded these findings clearly demonstrate that the seven isolates that proved to be resistant to commonly used antibiotics have the ability of forming biofilms.

Antibiofilm formation activity against isolate 03/17–16 of *V. cholerae* against various antibiotics like tetracycline, ampicillin, amoxicillin, cotrimoxazole, erythromycin and nalidixic acid was observed in all the antibiotics (Fig. 2). Against ampicillin, a concentration of 0.03125 mg ml^−1^ yielded significant biofilm formation inhibition (*P*=0.05), while for erythromycin obtained significant differences on the biofilm formation inhibition at a concentration of 0.5 mg ml^−1^ (*P*=0.0001), 0.25 mg ml^−1^ (*P*=0.001), 0.125 mg ml^−1^ (*P*=0.01). Nalidixic acid treatment yielded significant biofilm inhibition at concentrations of 0.5, 0.25, 0.125, 0.0625 (*P*=0.0001) and 0.03125 mg ml^−1^ (*P*=0.05). It is more worrying that tetracycline, which was the commonly used antibiotic in the outbreak, had less inhibitory effects against biofilm formation.

Antibiofilm formation activity against isolate 02/17–09 of *V. cholerae* against various antibiotics was seen in all the antibiotics (Fig. 3), with some significant differences at various dosages. Amoxicillin did not have good inhibitory effects against isolate 02/17–09. On the other hand, cotrimoxazole showed a reverse activity with high inhibitory effects being observed at low dosages as compared with higher concentrations.

In Fig. 4 above antibiofilm formation activity against isolate 04/17–13 of *V. cholerae* against various antibiotics was seen in all the antibiotics with most antibiotics producing significant differences as compared with the positive control at various dosages as shown in Fig. 4 above. With this test isolate also inhibitory activities were observed more at lower dosages as compared at high dosages in all antibiotics bioassayed.

Isolate 05/17–07 was more prone to the inhibitory effects of the various dosages of the various antibiotics as compared to other isolates. Significant differences were also observed in most of the antibiotics used compared with the positive control (Fig. 5).

Antibiofilm formation activity against isolate 05/17–03 of *V. cholerae* for various antibiotics was low (Fig. 6). Interestingly, the concentrations of 0.5, 0.25, 0.125, 0.0625 and 0.03125 mg ml^−1^ were able to inhibit biofilm formation much more as compared with the positive control.

Antibiofilm formation activity against isolate 06/17–14 of *V. cholerae* against various antibiotics was seen in all the antibiotics, with no significance differences on tetracycline at all the concentration as compared with the positive control (Fig. 7). The rest of the antibiotics produced significant antibiofilm activity. Erythromycin, tetracycline and nalidixic acid also showed significant inhibitory activity against this isolate 06/17–14 at lower concentrations.

### Detection of some virulence factors of *V. cholerae*

**Fig. 8.**
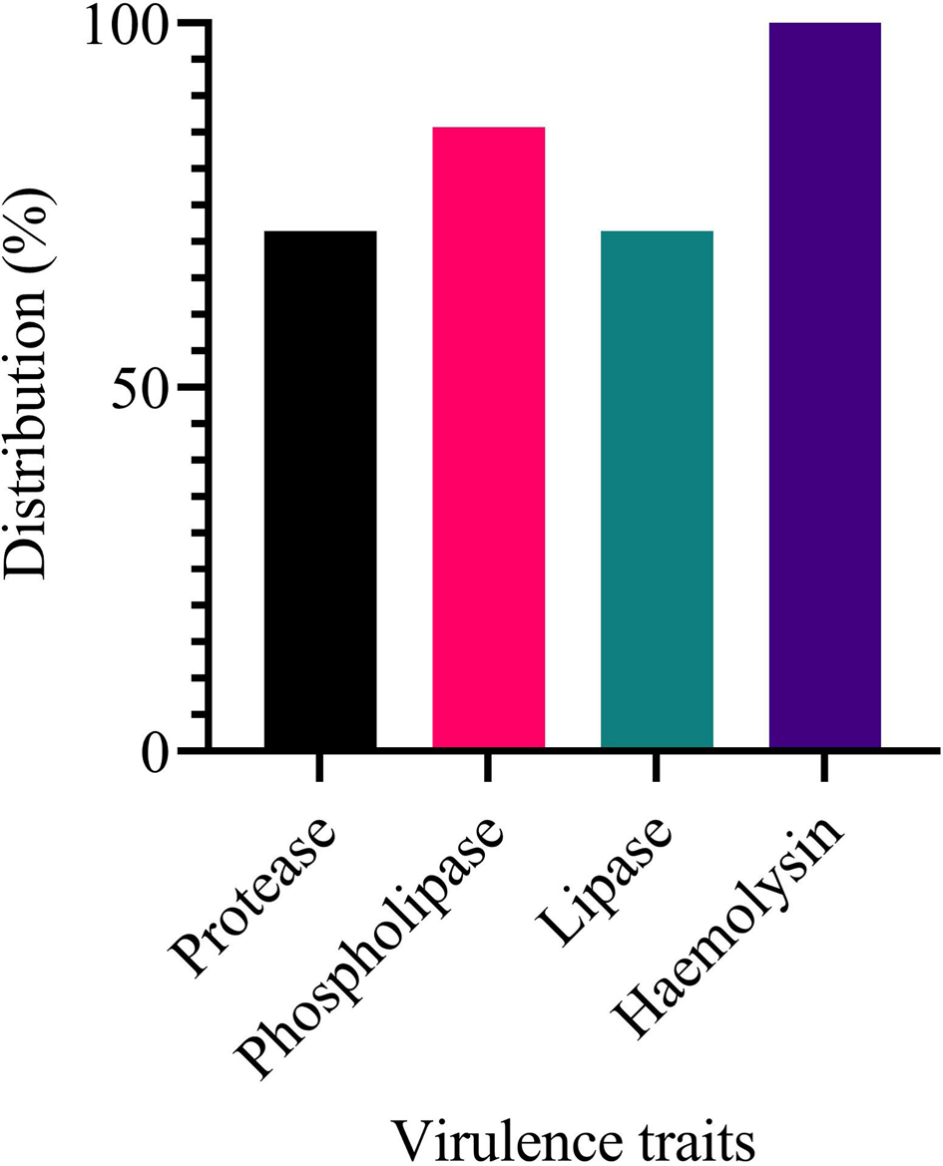
Showing the distribution of the seven isolates verses the various virulence traits they produced.

**Fig. 9.**
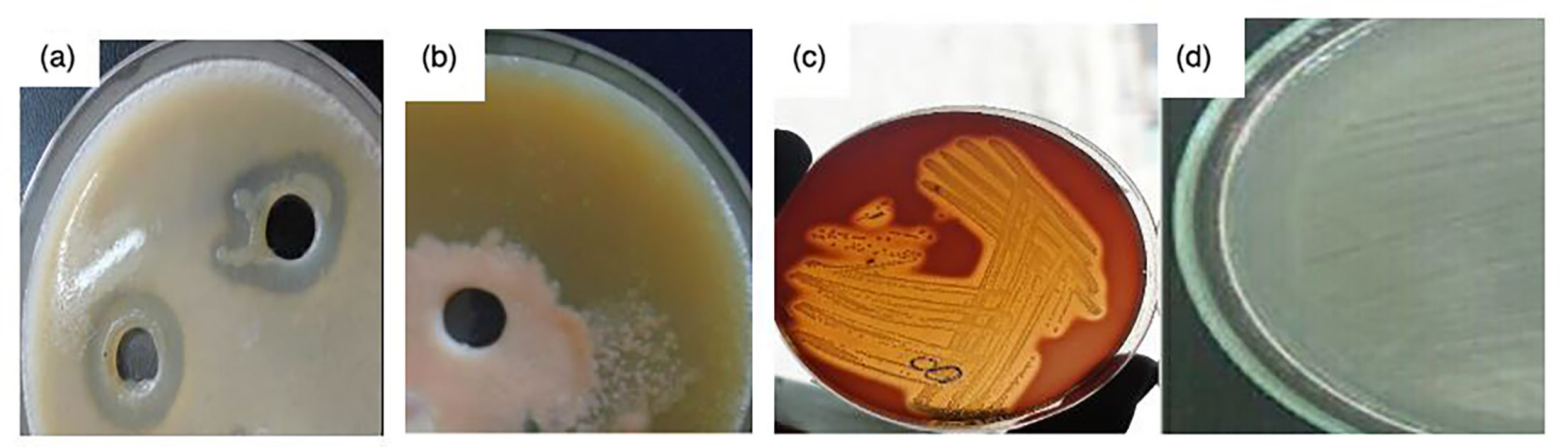
Representation of the various virulence traits produced by the *V. cholerae* isolates (a). The distribution of Protease enzyme production of *V. cholerae* (b). The Phospholipase enzyme production of *V. cholerae* (c). The haemolysin enzyme production of *V. cholerae –* haemolytic activity (d). Lipase enzyme production of *V. cholerae *.

The study investigated the production of various virulence enzymes like protease, phospholipase, lipase and haemolysin (Fig. 8) on the seven isolates, which were found to be resistant to all drugs as examined from our previous study [36]. It was revealed that 5/7 (71.42 %) of these isolates of *V. cholerae* produced protease enzyme (Fig. 9 – plate A; Fig. 8). Also, it was confirmed that six out seven isolates (85.71%) produce phospholipases (Fig. 9 – plate B; Fig. 8). Further findings also indicate that out of the seven isolates five of them (71.42%) had the ability to produce lipase (Fig. 9 – plate D; Fig. 8). Lastly, it was determined that all the seven isolates were able to produce the haemolysin by haemolysing the sheep red blood cells causing beta (β) haemolysis (Fig. 9 – plate C; Fig. 8) above. Haemolysin therefore was the most produced virulence factor by these isolates (100%) and it was followed by phospholipase (85.71%) and lastly lipase and protease (71.42%). Out of the seven isolates four isolates (03/17–16, 02/17–09, 04/17–13 and 06/17–07) produced all the virulence traits studied, two isolates (06/17–14 and 05/17–07) produced at least three virulence traits and one isolate (05/17–03) produced at least one of the virulence traits.

## Discussion

From this study, it was revealed that seven isolates that had been obtained from our previous study were resistant to various commonly used antibiotics [36] indeed possess various factors that enable them either to be resistant or induce various infections in humans. Some of these traits are controlled at the gene level and as such, they can be passed on from one bacteria cell to another through the pilli [27]. Antimicrobial drug resistance in *Vibrio* species for instance, may arise through mutation or through acquisition of resistance genes on mobile genetic elements like plasmids, transposons integrons and integrating conjugative elements [41]. Isolates analysed in this study possessed the class I integron and the SXT integrating conjugative element. Genetic elements like the class I integron (*inDS*) and the integrating conjugative elements such as SXT have been associated with the spread of genetic determinants, encoding for antimicrobial resistance in *V. cholerae* [42]. The SXT element has been reported to harbour genes encoding for resistance to chloramphenicol (encoded by *floR*), streptomycin (encoded by *strA and strB*), trimethoprim (encoded by *dfrA18*) and sulfamethoxazole (encoded by *sul2*) [43]. The class 1 integron has also been reported to harbour aminoglycoside-resistant gene cassettes in *V. cholerae* O1 isolates [44]. As such therefore, resistance to erythromycin in the analysed isolates could be attributed to the presence of the class 1 integron gene that was found to be present in some strains isolated.

The finding of susceptible isolates towards streptomycin but still amplifying a 383 bp fragment of the *strA* gene suggests that this gene is not an intrinsic feature of this family of integrase, but rather appears to have been inserted into these elements, becoming transmissible in bacterial populations, as reported by elsewhere [45]. Nalidixic acid resistance observed in this study could be attributed to mutations in the *gyrA* gene. Studies have reported *gyrA* gene mutations in fluoroquinolone-resistant clinical isolates of *V. cholerae* since it contains the active site tyrosine that forms a transient covalent intermediate with DNA hence making it to be resistance to the drug [46]. However, more studies need to be done to confirm this.

Some studies have also suggested that erythromycin and nalidixic acid do inhibit growth at high concentrations [12, 32] but from our study, it was deduced that they inhibit biofilm formation at lower concentrations. The same scenario was also observed in most antibiotics like tetracycline, amoxicillin, cotrimoxazole and others with such ‘Goldilocks’ effect. A possible explanation to the less activity observed at greater doses could be associated to the aggregation effects of the antibiotics at site of entry into bacteria cell especially at high dosages something that is not observed at lower dosages. It is likely that aggregation may favour biofilm formation as antibiotics struggle to reach at the point of action and hence bacteria will continue to thrive and hence form more biofilms [47]. This finding agrees with the previous studies on biofilm inhibitions by Taganna *et al*. [48], which showed higher biofilm inhibitory at lower dosage concentration against the positive control. However, it did not concur with the previous study on biofilm inhibition by [49], which showed that Erythromycin growth inhibitory at high concentration but also biofilm inhibitory at high concentration as compared with positive control [49]. On the other hand, tetracycline, ampicillin, amoxicillin and cotrimoxazole were found to inhibit biofilm formation at higher concentration in some isolates and against the positive control. However, it should be noted that they did not fully inhibit biofilm formation ability of the test isolates a clear indication that proper antibiotics should be used in management of conditions caused by these isolates.

Isolates 05/17–07 and 05/17–03 of *V. cholerae* seemed to be very sensitive to the antibiotics screened at various dosages. This clearly demonstrates that the biofilm formation by these isolates can easily be managed by these antibiotics. Since they proved to be resistant when they were subjected to antimicrobial tests following standard methods [36]. It is possible that these isolates could be possessing other mechanisms of activity against the antibiotics screened. One of such possible mechanisms could be the presence of multi-drug efflux pumps in them as they have been found to be present in some strains of *V. cholerae* [38]. This makes the bacteria to be resistant to antibacterial agents and other toxic compounds by a mechanism known as active efflux, where the integral membrane transporters known as drug efflux pumps, prevent the accumulation of drugs inside the bacterial cells [48].

Further, the study investigated the ability of these test strains to produce various virulence factors, which may play a role in their pathogenicity. Among the virulence traits examined include detection of proteases, lipases, haemolysin and phospholipase. The study revealed that 71.42 % of the isolates of *V. cholerae* produced protease. These findings confirm the findings of a previous study [50], which showed that most isolates were protease positive, and that protease enzyme have limited effect on the pathogenesis of this bacteria. Findings from the current study also agree with a study that documented that all isolates had the ability for protease production [14]. Proteases produced by *V. cholerae* have a critical role in pathogenicity, as they are responsible for hydrolysis of several physiologically important proteins such as mucin, fibronectin and lactoferrin [51]. It could also proteolytically activate cholera toxin A subunit, El Tor cytolycin and haemolysin, hence making this pathogen more virulent [52].

For phospholipases, 85.71 % of the isolates were found to be positive. These findings further confirm previous study findings where out of 20 isolates, 13 (67 %) isolates were found to have phospholipase production potential [13]. Our findings are also in tandem with Chung and Toh's [53] study findings for phospholipase presence. As previously mentioned [44], the role of this enzyme in the cholera disease by the release of arachidonic acid from the phospholipid found in the cell membranes of the lumen cells, this plays an important role in the prostaglandin E2 (PGE2) production, which is responsible for the increase of liquid secretion from the lumen cells. Therefore, its presence makes the *V. cholerae* isolate more virulent in watery diarrhoea production – a key symptom of cholera [54].

Also, out of the seven isolates five (71.42 %) had the ability to produce lipase. Our findings also concur with other studies, which showed that all isolates obtained in the study had the ability to produce lipase [14]. Lipases enzymes catalyse the hydrolysis of the ester bonds of triacylglycerols and may have a critical role in *V. cholerae* pathogenicity or nutrition acquisition. The production of an excess amount of lipases allows bacteria to penetrate fatty tissue with the consequent formation of abscesses [2]. The production of these enzymes by the isolates may reflect the presence of genetic organization of a discrete genetic element, which encodes three genes responsible to produce proteases, lipases and phospholipase. This organization could be a possible part of pathogenic island, encoding a product capable of damaging host cells and being involved in nutrient acquisition [51].

In this study, all isolates were able to produce the haemolysin. A finding that conforms with a previous study [50], where 100 % of isolates were haemolysin positive. As stated before, purified haemolysin can cause fluid accumulation [1], in contrast to the watery fluid produced in response to CT, the accumulated fluid produced in response to haemolysin with invariably bloody with mucous [1].

## Conclusion

From this study, it can be concluded that most clinical isolates had resistant genes and produced various virulence factors such as haemolysin, lipase, protease and phospholipase. As much as inhibitory effects were recorded, these findings clearly demonstrate that the isolates proved to be resistant to commonly used antibiotics and they do form biofilms. These findings, therefore, add value to our previous findings on these seven isolates that proved to be resistant against commonly used antibiotics in the management of cholera during a outbreak [36]. To the best of our knowledge, this is the first time such data has been documented from the outbreak region, that could help in combating future outbreak events.

## Supplementary Data

Supplementary material 1
