# Supplementary material for: Cholera outbreak: antibiofilm activity, profiling of antibiotic-resistant genes and virulence factors of toxigenic Vibrio cholerae isolates reveals concerning traits
Source: Access Microbiol. 2022 Mar 23;4(3):000324. doi: 10.1099/acmi.0.000324 (PMC9175979; doi:10.1099/acmi.0.000324)
Supplement: Supplementary material 1 [file acmi-4-0324-s001.pdf]

SUPPLEMENTARY DATA

Supplementary data S1: Biofilm formation after staining with 0.4% crystal violet solution

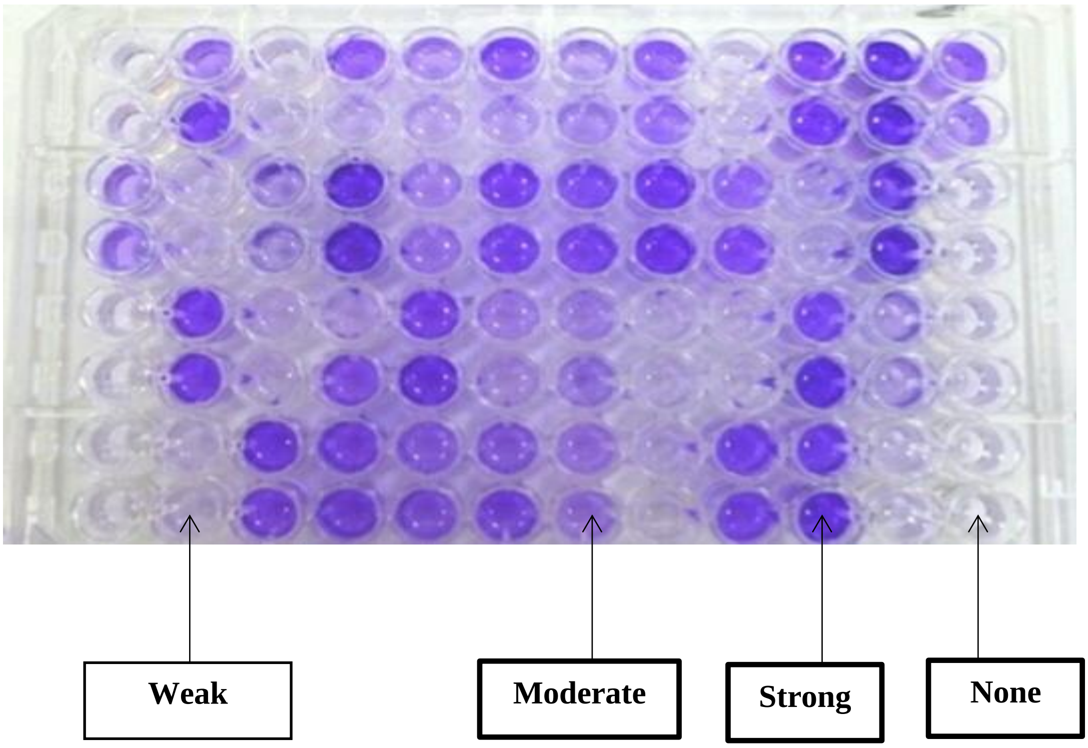

Supplementary data S2: Oligonucleotide primers, sequences, amplicons and conditions used in PCR assays in this study

Primer, genes, and sequence (5'- 3')	Amplicon Size (bp)	PCR conditions			Reference
		Melting	Annealing	Extension	
***inDs*, Class 1 integron**<br>F: CGGAATGGCCGAGATC<br>R: CAAGGTTCTGGACCACATTG	**380**	**95^0C, 1 min**	**58^0C, 1 min**	**72^0C, 1 min**	Dalsgaard *et al.* (1999)
***Int*, SXT element**<br>F: GCTGGATAGGTTAAGGGCGG<br>R: CTCTATGGGCACTGTCCACATTG	**592**	**95^0C, 1 min**	**54 ^{0}C, 1 min**	**72 ^{0}C, 1 min**	Hochhut *et al.* (2001).
***StrA*, Streptomycine Resistance**<br>F: TTGATGTGGTGTCCCGCAATG<br>R: CCAATCGCAGATAGAAGGCAA	**383**	**95^0C, 1 min**	**54 ^{0}C, 1 min**	**72 ^{0}C, 1 min**	Hochhut *et al.* (2001).
***Sul2*, Sulfamethoxale resistance**<br>F: AGGGGGCAGATGTGATCGAC<br>R: TGTGCGGATGAAGTCAGCTCC	**625**	**95^0C, 1 min**	**54 ^{0}C, 1 min**	**72 ^{0}C, 1 min**	Hochhut *et al.* (2001)
***TetA*-2000, tetracycline resistance**<br>F: GTAATTCTGAGCACTGTCGC<br>R: CTGCCTGGACAACATTGCTT	**950**	**95^0C, 1 min**	**58 ^{0}C, 1 min**	**72 ^{0}C, 1 min**	Yamai *et al.* (1997)
***ctxA*, Ct Subunit A**<br>F: TCAATTAGTTTGAGAAGTGC<br>R: TCAGATTGATAGCCTGAAAA	**564**	**95^0C, 1 min**	**54 ^{0}C, 1 min**	**72 ^{0}C, 1 min**	Unpublished
**toxR, toxR operon**<br>F: TTAACGCTGAATTACATTCA<br>R: TTAAGATTTACTGAACAGTA	**739**	**95^0C, 1 min**	**54 ^{0}C, 1 min**	**72 ^{0}C, 1 min**	Unpublished

Supplementary data S3: Rhan Media of Lipase Activity Assay preparation:

It was prepared by dissolving 5 g of K_2HP0_4, 5 g of $(NH_4)_2PO_4$, 1 g of Cacl2. $6H_2O$, 1g of $MgSO_4$. $7H_2O$, 0.001 g of $Fecl_2.6H_2O$, 0.001 g of NaCl, 20 g of agar powder and 5 ml of olive oil in 900 ml of distilled water. Then the volume was completed to 1000 ml, final pH was adjusted to 7.2, then autoclaved, cooled and poured in sterile Petri dishes and stored at 4˚C until to use. This media used to detect the ability of the bacteria to produce lipase (Rodina et al., 2018).

Supplementary data S4: Medium of Phospholipase Activity Assay:

Prepared by dissolving 2.4 g of nutrient agar in 100 ml of distilled water with 1 g of NaCl. After autoclaved and cooled to 50˚C, the addition egg yolk of one egg was done in a septical condition. Then mixed well and poured into sterile Petri dishes and stored at 4˚C until to be use between 24-48 hours (Dogan et al., 2003).
